# RAF1 promotes successful human cytomegalovirus replication and is regulated by AMPK-mediated phosphorylation during infection

**DOI:** 10.1128/jvi.01866-24

**Published:** 2025-02-04

**Authors:** Diana M. Dunn, Ludia J. Pack, Joshua C. Munger

**Affiliations:** 1 Department of Biochemistry and Biophysics, University of Rochester 6923 , Rochester, New York, USA; University of Virginia, Charlottesville, Virginia, USA

**Keywords:** HCMV, cytomegalovirus, RAF1, AMPK

## Abstract

RAF1 is a key player in growth factor receptor signaling, which has been linked to multiple viral infections, including human cytomegalovirus (HCMV) infection. Although HCMV remains latent in most individuals, it can cause acute infection in immunocompromised populations, such as transplant recipients, neonates, and cancer patients. Current treatments are suboptimal, highlighting the need for novel therapies. Multiple points in the growth factor signaling pathway are important for HCMV infection, but the relationship between HCMV and RAF1, a component of the mitogen-activated protein kinase (MAPK) cascade, is not well understood. The AMP-activated protein kinase (AMPK) is a known regulator of RAF1, and AMPK activity is induced by HCMV infection, which is important for productive HCMV replication. Our data indicate that HCMV infection induces AMPK-specific changes in RAF1 protein phosphorylation, including increasing phosphorylation at RAF1-Ser621, a known AMPK phospho-site, which results in increased binding to the 14-3-3 scaffolding protein, an important aspect of RAF1 protein activation. Inhibition of RAF1, either pharmacologically or via shRNA or CRISPR-mediated targeting, inhibits viral replication and spread in both fibroblasts and epithelial cells. Collectively, our data indicate that HCMV infection and AMPK activation modulate RAF1 activity, which is important for viral replication.

**IMPORTANCE:**

Human cytomegalovirus (HCMV) infection is a widespread infection impacting approximately 60–90% of the global population. Although latent in healthy individuals, acute infection in immunocompromised populations, such as neonates, transplant recipients, and cancer patients, can result in retinal and gastrointestinal problems, hearing loss, and even death. Current antivirals are suboptimal due to the development of viral resistance or toxicity in patients, highlighting the need for novel treatments. Our research suggests a new potential target, RAF1, which is a regulator of cellular growth and proliferation. We find that RAF1 is phosphorylated by AMP-activated protein kinase, and that inhibition of RAF1 negatively impacts viral infection. Furthermore, drugs currently used to treat certain cancers also inhibit RAF1 and may have an additional anti-HCMV therapeutic effect in HCMV-susceptible cancer patients.

## INTRODUCTION

Human cytomegalovirus (HCMV) is a prevalent virus infecting approximately 60–90% of the global population (1). Although it remains latent in healthy individuals, it can lead to damaging acute infection in immunocompromised individuals, such as neonates (2, 3), transplant recipients (4, 5), those undergoing immunosuppressive anti-cancer therapies (6–10), and those receiving anti-AIDS therapies (11). Current treatments for HCMV are suboptimal, often resulting in drug resistance and unwanted side effects (12–15) and underscoring the need to develop novel therapeutics to treat acute HCMV infection.

HCMV is a beta herpes virus with a large double-stranded DNA genome encoding for upwards of 200 open reading frames with the potential to express hundreds of viral proteins, many of which have unknown functions or expression patterns (16). HCMV, like many other viruses, also hijacks and relies on various host factors for successful infection (17). One of these host proteins is AMP-activated protein kinase (AMPK), a central metabolic stress regulator, whose expression and activity are induced by HCMV infection to support virally induced metabolic remodeling and promote viral replication (18, 19).

**Fig 1:**
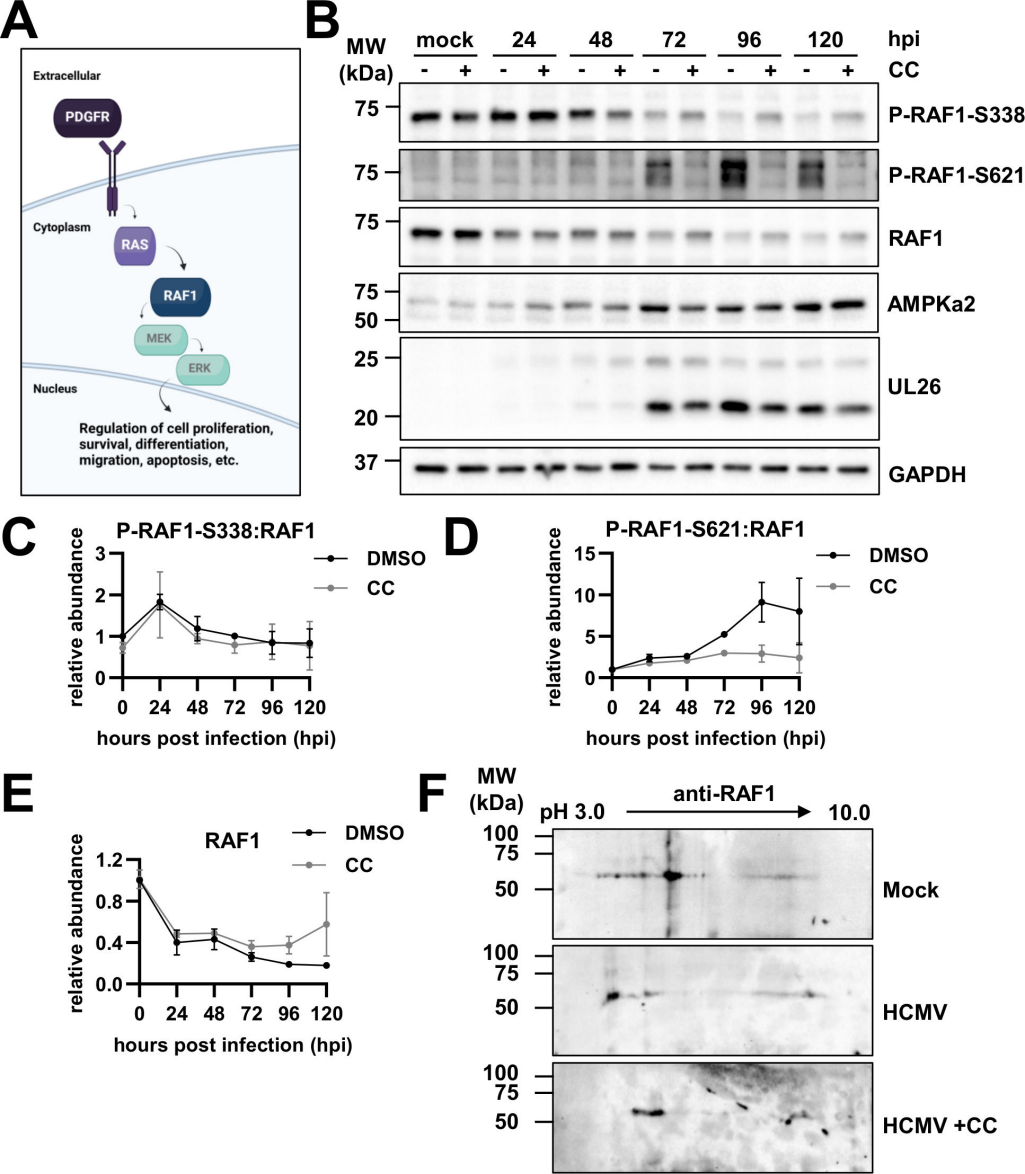
Human cytomegalovirus (HCMV) infection induces the AMP-activated protein kinase-specific phosphorylation of RAF1. (**A**) Schematic depicting the RAF1 signaling pathway. (**B**) Western blot analysis of RAF1 protein over the course of infection (120 h) in the absence and presence of 5 µM compound C (CC) in MRC5 fibroblasts. Glyceraldehyde-3-phosphate dehydrogenase (GAPDH) was used as a loading control, and UL26 is a control for successful viral infection. (**C–E**) Quantification of Western blots in (**B**), where 0 h post-infection (hpi) is representative of mock infection (avg ± standard error of the mean, *n* = 2), comparing phospho-RAF1 to total RAF1 for (**C**) P-RAF1-S338 and (**D**) P-RAF1-S621, and (**E**) total RAF1 levels relative to GAPDH. (**F**) Two-dimensional gel electrophoresis of RAF1 protein during mock infection and HCMV infection with or without CC treatment, MOI = 3.0 at 48 hpi in MRC5 fibroblasts (*n* = 1).

Growth factor signaling has been linked to the productive infection of multiple viruses, including influenza (20), Zika (21), coronaviruses (22), and cytomegalovirus (23). Growth factor receptors facilitate viral entry, internalization, replication, and egress (24). A typical growth factor signaling pathway involves binding to receptor tyrosine kinases, such as platelet-derived growth factor receptor (PDGFR) or epidermal growth factor receptor (EGFR), which results in downstream activation of RAS and the subsequent cascade of RAF1, MEK, and ERK leading to regulation of multiple cellular processes, including proliferation, survival, differentiation, migration, apoptosis, and more (25, 26) (Fig. 1A).

Human cytomegalovirus relies on multiple stages of growth factor signaling to support productive infection (23). For example, PDGFR and EGFR not only facilitate viral entry (27, 28) but also signal downstream cellular pathways to sustain viral signaling, trafficking, gene transcription, and latency (29–31). Soluble derivatives of PDGFR have been demonstrated to bind and neutralize free HCMV particles, thus reducing viral entry into cells (32, 33). Furthermore, inhibition of PDGFR by imatinib or nilotinib can inhibit viral entry and HCMV infection when cells are pretreated with these drugs (34). Likewise, treatment of cells with other various broad-spectrum MAPK inhibitors attenuates HCMV replication (35, 36). However, it is well known that HCMV is unable to replicate in transformed cell lines, and RAS/SV40-mediated transformation of human cells leads to the inability of HCMV to replicate (37). Collectively, these data indicate a complex relationship between HCMV and growth factor receptor signaling.

RAF1, also known as CRAF, is a heavily phosphorylated mitogen-activated protein kinase kinase kinase, and the dynamic nature of its phosphorylation plays a complicated role in its kinase activity (38). Phosphorylation at Ser338 activates the RAF1 protein (39), and RAF1-Ser621 phosphorylation is necessary for its activation and stability achieved through binding to the scaffold protein and activating co-factor, 14-3-3 (40–44). The main site within RAF1 that is targeted by AMPK is Ser621, although Ser259 is also putatively modified by AMPK, as well as other kinases, including PKCA and RAF1 itself (45–47). Crosstalk between these phosphorylation events and others within RAF1 can impact multiple aspects of the protein, including its cellular location, stability, and activity (38, 43, 48).

Here, we explore the phosphorylation of RAF1 and its contributions to HCMV infection. We find that HCMV induces AMPK-specific phosphorylation of RAF1 and in turn increases RAF1 binding to the scaffolding protein, 14-3-3. Pharmaceutical inhibition of RAF1 results in reduced viral DNA and protein accumulation and attenuates infectious virion production. Furthermore, RAF1 knockdown by shRNA or knockout by CRISPR results in decreased viral titers and reduced cell-to-cell spread.

## RESULTS

### HCMV infection induces AMPK-specific phosphorylation of RAF1

To evaluate the effects of AMPK on RAF1, we first assessed the specific phosphorylation of RAF1-S338 and -S621 by western blot during infection in the presence of DMSO or AMPK inhibitor Compound C (CC) (Fig. 1B). Previous work has shown that Compound C inhibits viral infection (18, 19, 49). In Fig. 1, there is an early increase in activated RAF1 phosphorylated at S338, which dropped as infection proceeded (Fig. 1B and C). The ratio of RAF1-S338 phosphorylation to the total remained unchanged in DMSO versus Compound C-treated samples at each time point, suggesting that AMPK does not impact the phosphorylation of RAF1 at this site (Fig. 1B and C). In contrast, the level of RAF1-S621 phosphorylation increased substantially during infection, which was blocked by treatment with Compound C (Fig. 1B and D). Notably, RAF1 levels dropped by approximately 50% relative to mock at 24 h post-infection (hpi) and remained there until 48 hpi, then decreased further as infection progressed (Fig. 1B and E). Although total RAF1 levels were marginally higher with Compound C treatment compared to DMSO control at each time point, they were not fully restored to mock or early infection levels (Fig. 1B and E). The increase in RAF1-S621 phosphorylation and the loss of total RAF1 expression mirrored increases in AMPKa2 protein levels, which have previously been found to be important for HCMV infection (Fig. 1B) (18, 19).

To further explore how HCMV and AMPK impacted the post-translational modifications of RAF1, we analyzed RAF1 via two-dimensional (2D) gel electrophoresis (Fig. 1F). During HCMV infection, RAF1 populations had a significantly higher negative charge relative to RAF1 during mock infection, resulting in a shift toward pH 3.0, consistent with increased phosphorylation (Fig. 1F). The addition of Compound C during infection resulted in less negatively charged RAF1, shifting the RAF1 isoforms toward the more basic (pH 10.0) cathode and suggesting substantially decreased phosphorylation, although not back to the same position of mock-infected cells (Fig. 1F). These results indicate that AMPK likely plays an important role in regulating RAF1 phosphorylation during HCMV infection.

**Fig 2:**
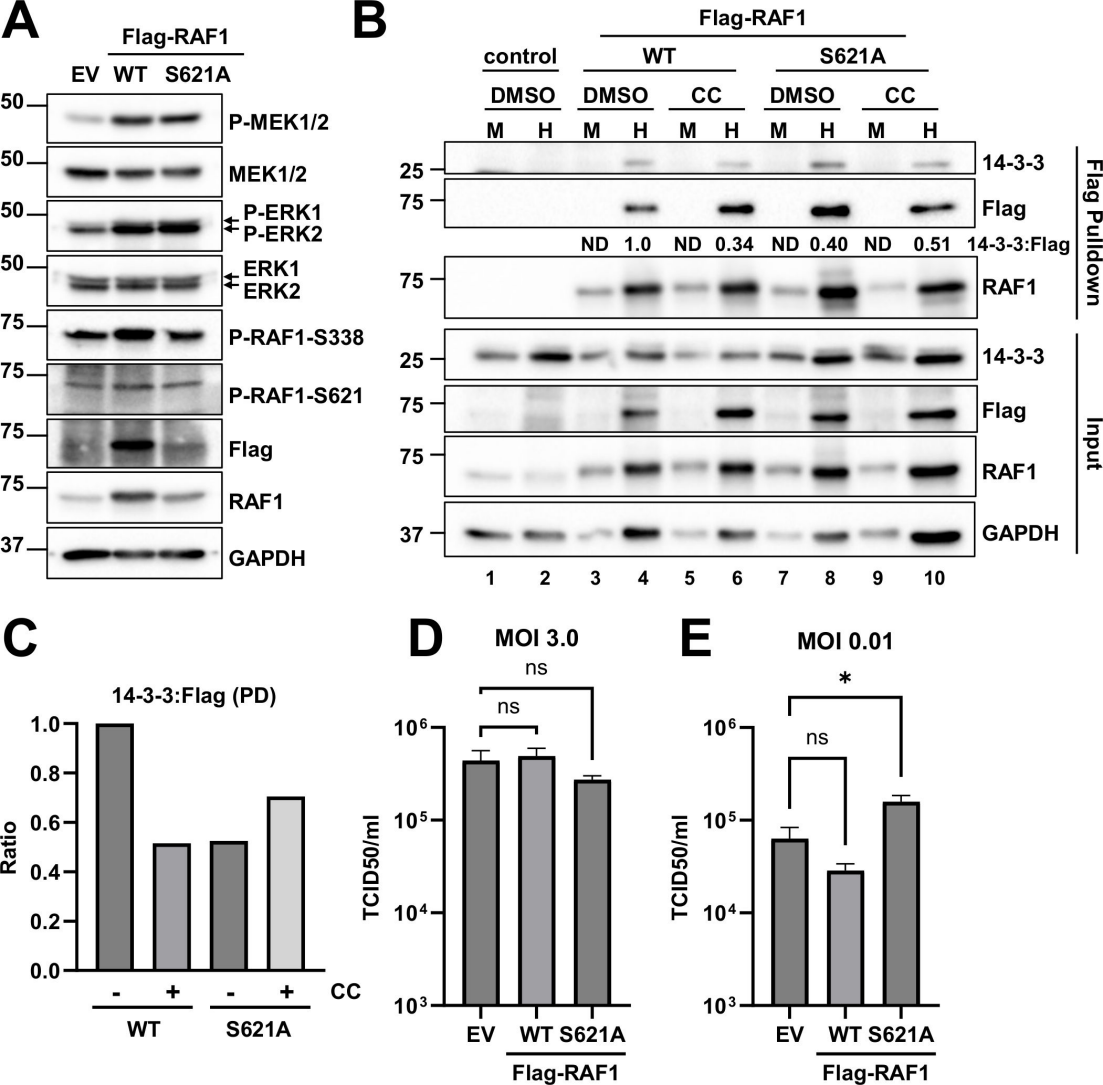
Phosphorylation of S621 enhances RAF1 binding to 14-3-3 during HCMV infection. (**A**) Overexpression of Flag-RAF1-WT (wild type) or Flag-RAF1-S621A via lentiviral transduction were compared to control MRC5 fibroblasts and assessed for protein expression by western blot using antibodies specific to Flag, RAF1, and S621- or S338-phosphorylated RAF1 and total or phospho-MEK1/2 and -ERK1/2 (arrows indicate doublet). Glyceraldehyde-3-phosphate dehydrogenase (GAPDH) was used as a loading control (*n* = 1). (**B**) Empty vector (EV), Flag-RAF1-WT, and -S621A transduced cells were mock (**M**)- or HCMV (**H**)-infected with AD169-WT at an MOI of 3.0 for 72 h in the presence or absence of 5 µM Compound C (CC) prior to Flag pulldown and co-precipitation of endogenous 14-3-3. Numbers indicate the ratio of 14-3-3 to Flag protein pulled down in the representative blot. (**C**) Ratios of 14-3-3 to Flag pulled down during HCMV infection lanes in (**B**) were quantified along with a second blot (not shown) (avg, *n* = 2). (**D**) Cells were infected at an MOI of 3.0 for 120 h or (**E**) at an MOI of 0.01 for 9 days (avg ±SEM, *n* = 4), and viral titers were assessed by TCID50. Significance was established by a one-way analysis of variance test, followed by Tukey’s multiple-comparisons test.

Given the observation that HCMV induced RAF1-S621 phosphorylation (Fig. 1), we wanted to explore the impact of the expression of a RAF1 allele that is non-phosphorylatable at this site. Toward this end, Flag-RAF1-WT (wild type) or Flag-RAF1-S621A was constructed and delivered to fibroblasts via lentiviral transduction (Fig. 2). RAF1 protein levels were elevated compared to empty vector control cells (Fig. 2A). In addition, Flag-RAF1-WT accumulated to higher levels compared to Flag-RAF1-S621A (Fig. 2A), which could be expected as S621 phosphorylation is important for RAF1 stability (42). Overexpression of Flag-RAF1-WT also increased S338 phosphorylation (Fig. 2A), suggesting that overexpression of RAF1 induces its activity in MRC5 fibroblasts. Expressions of P-ERK and P-MEK were also elevated, indicative of an active RAF1 pathway, even in the presence of the S621A mutation. Phosphorylation of S621 was slightly elevated in the WT-RAF1 cell line but not in the cells expressing RAF1-S621A, confirming that this mutation attenuates phosphorylation at this site (Fig. 2A).

RAF1-S621 phosphorylation is a prerequisite for its activation via binding to the scaffold protein, 14-3-3 (40–44). We, therefore, assessed the interaction between RAF1 and 14-3-3 during infection in the absence or presence of the AMPK inhibitor, Compound C (CC). Cells were transduced with Flag-RAF1-WT or Flag-RAF1-S621A expression constructs and subsequently infected (MOI 3.0). At 72 h post-infection, the Flag-fused RAF1 proteins were purified via Flag-antibody affinity and analyzed for co-precipitation with 14-3-3 (Fig. 2B). The ratio of co-precipitated 14-3-3 and Flag-fused RAF1 is indicative of their relative association during infection. HCMV infection precipitated significant amounts of 14-3-3 in cells transduced with WT-RAF1, which was not detectable in uninfected cells (Fig. 2B and C). The co-precipitated 14-3-3:Flag ratio was reduced when the RAF1-S621A allele was expressed during infection (Fig. 2B and C), suggesting reduced 14-3-3 association, which is consistent with previous reports indicating that S621 phosphorylation is important for 14-3-3 binding (40–44). Compound C treatment reduced the 14-3-3:WT-RAF1 ratio during HCMV infection but did not appreciably change the ratio of co-precipitated 14-3-3:RAF1-S621A (Fig. 2B and C), which is again consistent with AMPK activity modulating RAF1–14-3-3 association via serine 621 phosphorylation. These data suggest that AMPK-mediated phosphorylation of RAF1 increases its binding to 14-3-3 during HCMV infection, thus regulating RAF1 stabilization and activation.

Given that growth factor signaling has generally been found to be important for HCMV infection (27, 28), we hypothesize that RAF1 activation supports HCMV infection. While the overexpression of either RAF1-WT or RAF1-S621A increased the downstream phosphorylation of RAF1 targets, including MEK and ERK (Fig. 2A), we wanted to determine if the expression of these RAF1 variants differentially impacted infection. At a high MOI (3.0), neither the expression of WT nor the mutant RAF1 allele significantly impacted viral titers (Fig. 2D). At a low MOI (0.01), there was a slight but statistically significant increase in the production of HCMV progeny after expressing the RAF1 mutant (Fig. 2E). These data suggest that overexpression of either WT- or S621A-mutated RAF1 does not substantially impact HCMV infection.

### Pharmacological inhibition of RAF1 reduces HCMV infection

**Fig 3:**
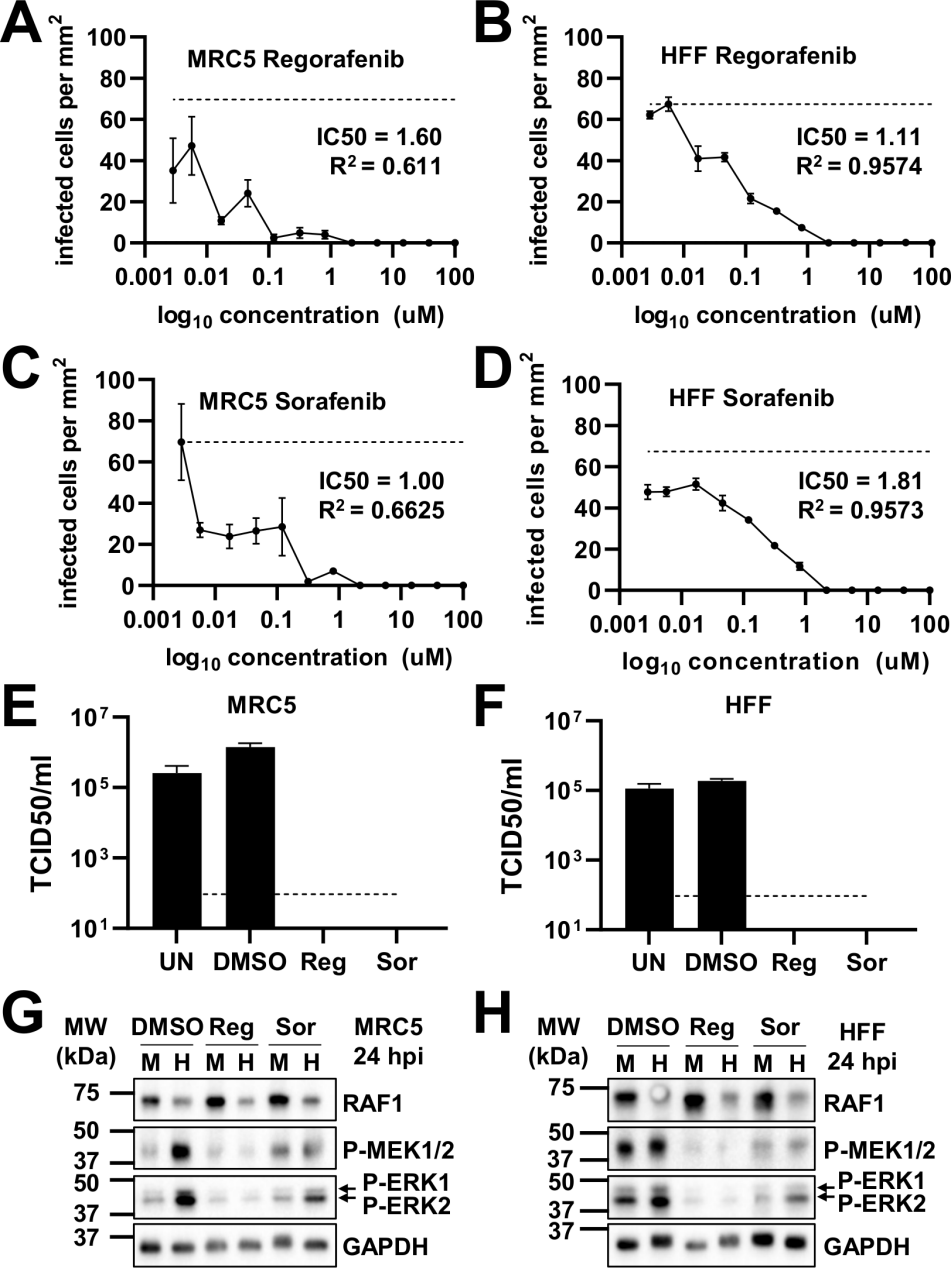
Pharmacological inhibition of the RAF1 pathway inhibits HCMV infection. (**A, B**) Regorafenib (Reg) or (**C, D**) sorafenib (Sor) treated (**A, C**) MRC5 or (**B, D**) HFF fibroblasts were treated with the indicated concentration of drugs or DMSO control at the time of infection. (**A–D**) Cells were infected at an MOI of 0.1 with AD169-GFP for 5 days, then fixed. GFP-positive cells were counted per area of the well and plotted against drug concentration [avg ± standard error of the mean (SEM), *n* = 3]. IC50 values were calculated. The dotted line indicates the average maximal GFP in untreated cells. (**E–H**) Fibroblasts were infected at an MOI of 3.0 in the presence of DMSO or 2.17 µM regorafenib or sorafenib added at the time of viral adsorption. UN is an untreated control. Viral titers from (**E**) MRC5 or (**F**) HFF cells were assessed by TCID50 (avg ± SEM, *n* = 3) 120 hpi. The dotted line indicates the limit of detection. In (**G**) MRC5 or (**H**) HFF cells, at 24 h post-infection (hpi), phosphorylation of MEK1/2 and ERK1/2 (arrows indicate doublet) was assessed by western blot (*n* = 1).

**Fig 4:**
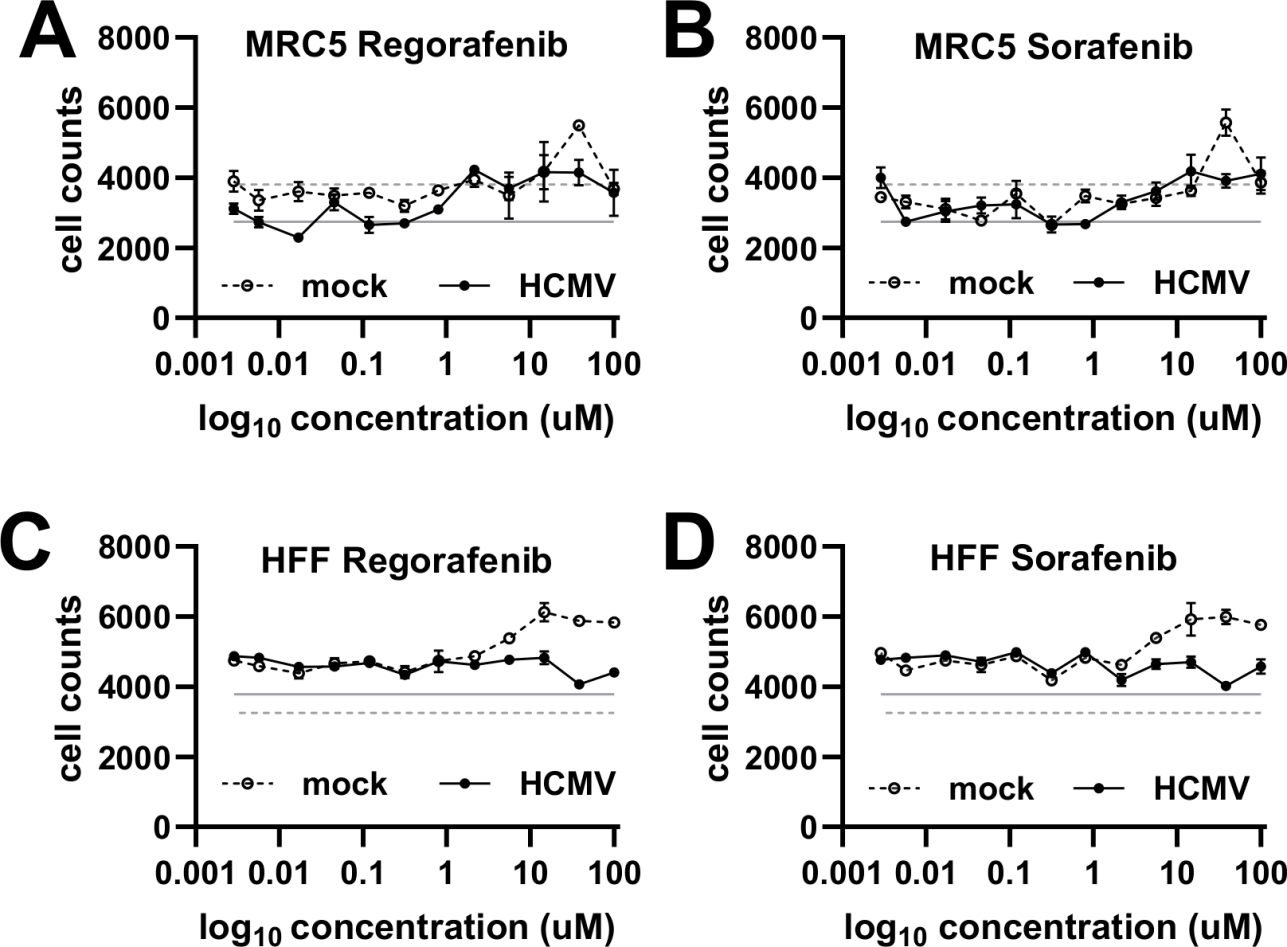
Pharmacological inhibition of the RAF1 pathway does not impact fibroblast cell counts. (**A, B**) MRC5 or (**C, D**) HFF fibroblasts were treated with the indicated concentration of (**A, C**) regorafenib or (**B, D**) sorafenib at the time of infection, AD169-GFP, MOI = 0.1, fixed on Day 5. (**A–D**) Hoechst-stained nuclei were counted and plotted against the concentration (avg ± standard error of the mean, *n* = 3). The solid gray line indicates the average number of nuclei in untreated HCMV-infected cells, while the dotted gray line indicates the average number of nuclei in untreated mock-infected cells.

We next evaluated the effect of RAF1 inhibition on viral replication. Regorafenib and sorafenib are RAF1-specific inhibitors, although they also inhibit other kinases in the RAF1 pathway, albeit with higher IC50s (Table 1) (50, 51). To examine how HCMV replicates in the presence of these compounds, we monitored HCMV infection in two fibroblast cell lines, MRC5 and HFF, in a dose–response to these compounds (Fig. 3). After 5 days, cells positive for GFP were counted and plotted per area of cell growth against compound concentration (Fig. 3A through D). Both sorafenib and regorafenib limited viral replication, as indicated by the loss of GFP-positive cells at 2.17 µM (Fig. 3A through D). These results were consistent with a previous report using sorafenib (36). We subsequently examined how these compounds impact the production of viral progeny and found that the amount of infectious virus produced in their presence was below the limit of detection ( Fig. 3E and F). As expected, treatment with either sorafenib or regorafenib substantially reduced the phosphorylation of downstream RAF1 targets, including P-MEK and P-ERK ( Fig. 3G and H). Furthermore, the inhibitors were generally well-tolerated in mock- and HCMV-infected cells (Fig. 4A through D ). Together, these data indicate that inhibition of the RAF1 pathway can inhibit viral infection and spread without impacting cell viability.

**TABLE 1:** Reported IC50s of regorafenib and sorafenib to recombinant RAF pathway proteins (50, 51)

RAF family protein	IC50—Regorafenib	IC50—Sorafenib
RAF1	2.5 nM	6 nM
BRAF	28 nM	22 nM
BRAF (V600E)	19 nM	38 nM
PDGFRB	22 nM	57 nM

### Inhibition of RAF1 negatively impacts viral DNA and protein accumulation

**Fig 5:**
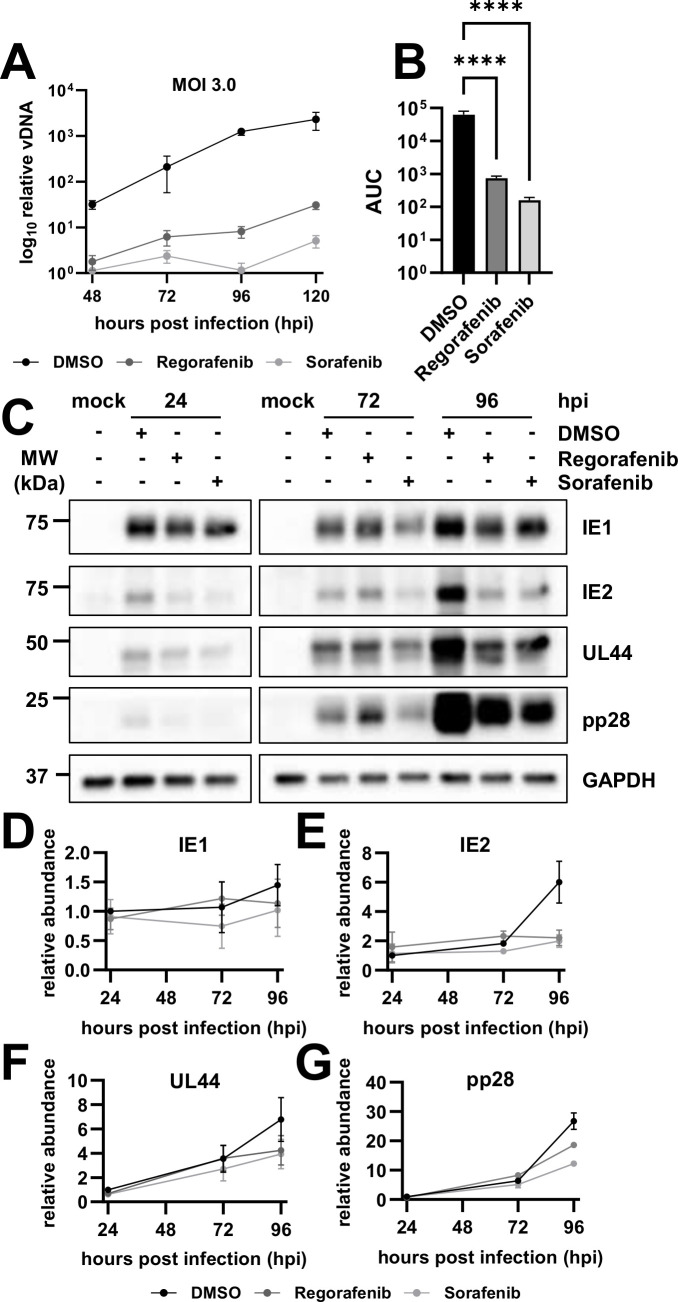
Inhibition of the RAF1 pathway negatively impacts viral DNA and protein accumulation. (**A–C**) MRC5 fibroblasts were treated with 2.17 µM of regorafenib or sorafenib or DMSO control at the time of infection with AD169 at an MOI of 3.0 for the indicated amount of time. (**A**) Viral DNA was quantified by RT-qPCR of the viral gene, IE1 [avg ± standard deviation (SD), *n* = 12]. (**B**) Samples in (**A**) were compared by area under the curve (AUC) to show the significance between treatments over the course of infection (avg ± SD, *n* = 12). Significance was established by a one-way analysis of variance test, followed by Tukey’s multiple-comparison test. (**C**) Viral protein expression levels at 24, 72, and 96 h post-infection (hpi) were assessed by western blot (*n* = 2). Glyceraldehyde-3-phosphate dehydrogenase (GAPDH) was used as a loading control. (**D–G**) Quantification of western blots in (**C**) (avg ± standard error of the mean, *n* = 2) normalized to GAPDH and DMSO, 24 hpi sample for viral proteins (**D**) IE1, (**E**) IE2, (**F**) UL44, and (**G**) pp28.

To elucidate how RAF1 inhibition impacts the viral life cycle, we first measured the viral DNA accumulation from 48 to 120 hpi (Fig. 5A). Significantly less viral DNA were accumulated over the course of infection when cells were treated with regorafenib and sorafenib relative to DMSO control, as indicated by the area under the curve for each sample (Fig. 5B). Regorafenib and sorafenib treatment modestly reduced the IE1, IE2, and UL44 protein levels at 24 and 72 hpi, but by 96 hpi, inhibitor treatment resulted in a substantially less accumulation of these proteins, as well as pp28, a late protein (Fig. 5C through G). The modest impact of RAF1 inhibition on immediate early gene expression at the start of infection suggests that RAF1 activity is not necessary for viral entry; rather, it indicates that RAF1 is important for post-entry early events of infection leading to DNA replication.

### Knockdown or partial knockout of RAF1 reduces HCMV infection

**Fig 6:**
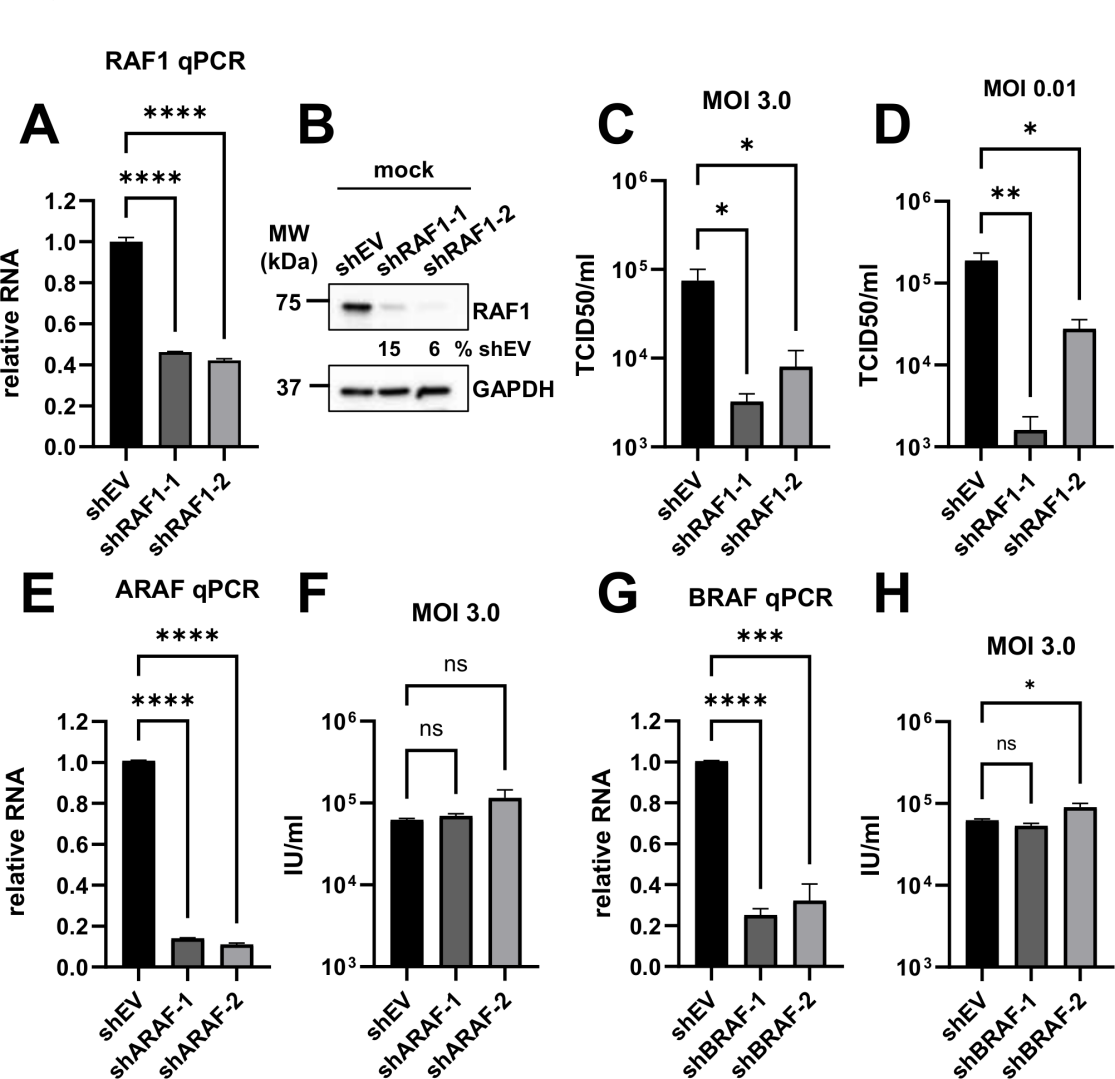
RAF1 shRNA-mediated knockdown inhibits HCMV infection. (**A–H**) Indicated shRNA guides were delivered via lentiviral delivery in HFF fibroblast cells. (**A–D**) RAF1 shRNA (shRAF1) were assessed for (**A**) knockdown efficiency in uninfected fibroblasts by RT-qPCR [avg ± standard error of the mean (SEM), *n* = 3 or 4], (**B**) protein expression was assessed via western blot (*n* = 1), and (**C–D**) viral titer was measured by TCID50 (avg ± SEM, *n* = 4) at (**C**) an MOI of 3.0 at 120 h post-infection, or (**D**) an MOI of 0.01 at 10 days post-infection. (**E–F**) ARAF shRNA (shARAF) were assessed for (**E**) RNA levels via RT-qPCR and (**F**) viral titers via the GFP expression of cells infected at an MOI of 3.0 at 120 h post-infection. (**G–H**) BRAF shRNA (shBRAF) were assessed for (**G**) RNA levels via RT-qPCR and (**H**) viral titers via GFP expression of cells infected at an MOI of 3.0 at 120 h post-infection. (**A, E, G**) RNA levels were normalized to glyceraldehyde-3-phosphate dehydrogenase (GAPDH) and shRNA empty vector (shEV) control in all RT-qPCR experiments. Significance was established by a one-way analysis of variance test, followed by Tukey’s multiple-comparison test.

Given that sorafenib and regorafenib can inhibit other kinases in the RAF1 pathway, including BRAF (Table 1), we employed other methods to analyze RAF1’s potential contributions to HCMV infection. First, we targeted RAF1 with two different shRNA constructs in HFF cells. The knockdown efficiency was assessed by qPCR, which indicated 54 and 58% losses in the expression of RAF1 RNA compared to the empty vector (shEV) shRNA control (Fig. 6A). At the protein level, RAF1 was reduced by 85 and 94% compared to shEV cells (Fig. 6B). At a high MOI (3.0), these constructs reduced HCMV infection by approximately 10-fold (Fig. 6C). A similar replication defect was observed at a low MOI (0.01) (Fig. 6D), highlighting the importance of RAF1 for HCMV infection.

To explore the possible contributions that the other RAF proteins make toward viral infection, ARAF and BRAF were targeted via lentiviral-delivered shRNA (Fig. 6E through H). With an 86 and 89% reduction in the expression of ARAF RNA (Fig. 6E), viral titers were not significantly impacted by the reduced ARAF levels (Fig. 6F). The RNA expression of BRAF was reduced by 75 and 68% (Fig. 6G), which also did not significantly decrease the viral titer compared to the shEV control (Fig. 6H). These data suggest that RAF1 is the RAF kinase most relevant to HCMV infection.

**Fig 7:**
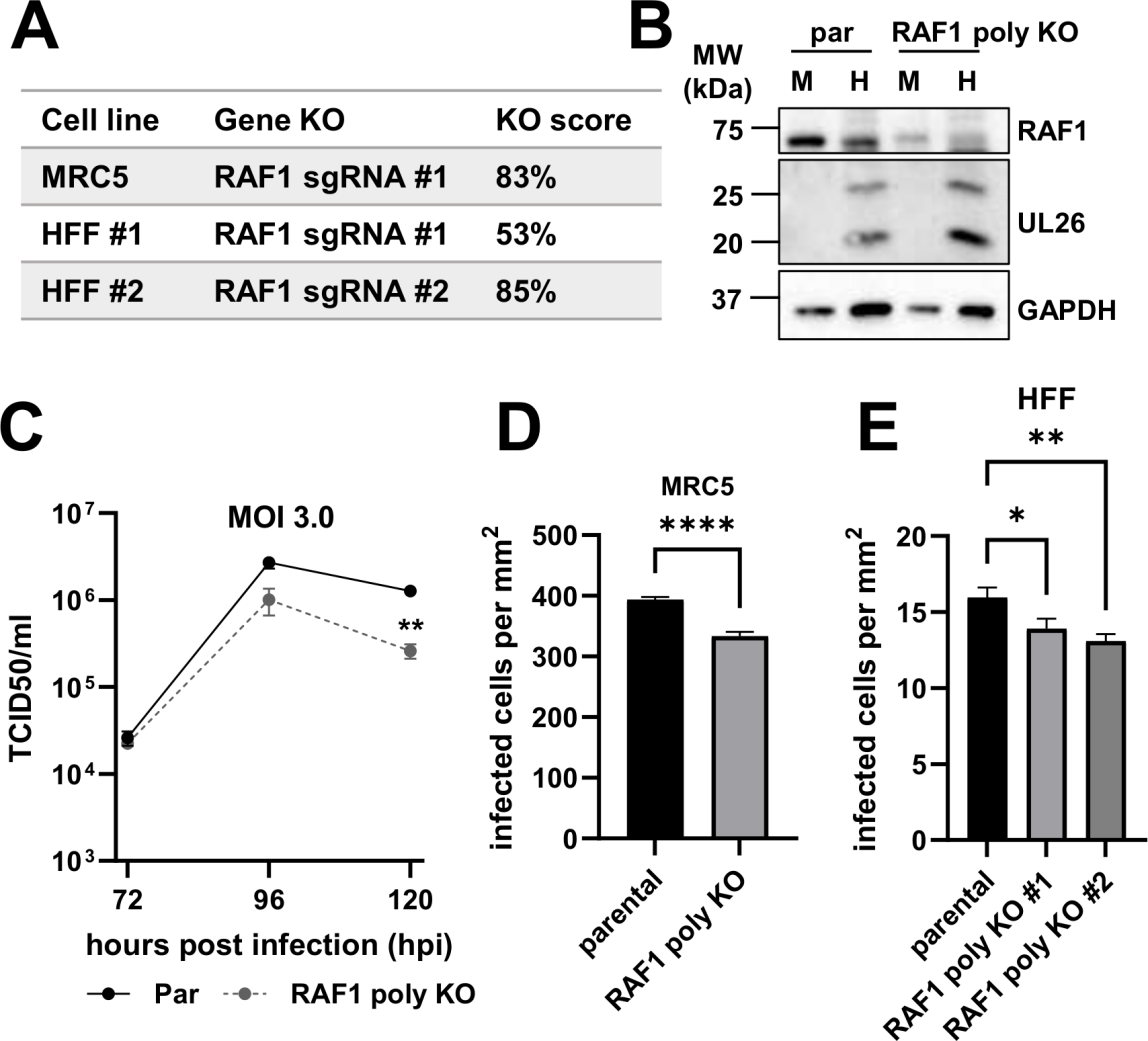
CRISPR-mediated knockout of RAF1 attenuates cell-to-cell spread of HCMV. (**A**) Knockout (KO) scores for CRISPR RAF1 KO cell lines generated. (**B–D**) MRC5 parental (par) and RAF1 polyclonal CRISPR knockout (poly KO) cells were infected at an MOI of 3.0 for (**B**) 96 h, and protein expression was assessed by western blot during mock (**M**) or HCMV (**H**) infection (*n* = 1). UL26 is a control for successful viral infection, and glyceraldehyde-3-phosphate dehydrogenase (GAPDH) is a protein loading control. (**C**) At 72, 96, and 120 h post infection (hpi), viral titers were assessed by TCID50 [avg ± standard error of the mean (SEM), *n* = 2]. Significance is based on Student’s *t*-test at 120 hpi. (**D**) MRC5 (avg ± SEM, *n* = 72) and (**E**) HFF (avg ± SEM, *n* = 48), parental, and RAF1 polyclonal CRISPR knockout cell lines were infected with AD169-GFP at an MOI of 0.05 for 10 days. Infected cells per area of the well were counted via GFP expression, and significance was established by a one-way analysis of variance test, followed by Tukey’s multiple-comparison test.

We also generated polyclonal RAF1 knockout (KO) cell lines via transfection of ribonucleoprotein-CAS9 and RAF1-specific guide RNAs (sgRNA) into MRC5 and HFF cells. The RAF1 KO score was 83% in the MRC5 cell line and 53 and 85% in the HFF cell lines (Fig. 7A). Consistent with these findings, the CRISPR targeting of RAF1 resulted in approximately a 65% reduction in RAF1 protein levels in the mock-infected MRC5 KO cells (Fig. 7B). During infection, the CRISPR-mediated inactivation of RAF1 in the MRC5 cells did not impact the accumulation of viral protein, UL26 (Fig. 7B). However, we observed that RAF1 KO in MRC5 cells reduced viral titers relative to parental cells at 120 h post-infection (Fig. 7C). We also assessed how the RAF1 knockout impacted the HCMV cell-to-cell spread in both RAF1 KO MRC5 and HFF cells. After infection at an MOI of 0.05 and incubation for 10 days, the number of GFP-positive cells was significantly lower in the RAF1 KO cells (Fig. 7D and E). Given the slight impact that sorafenib and regorafenib treatment had on the IE1/2 expression, we also explored the expression of IE1 and IE2 in the RAF1 shRNA and RAF1 CRISPR cell lines (see Fig. S1 at https://doi.org/10.6084/m9.figshare.27981740). Loss of RAF1 in either case did not negatively impact immediate early gene expression, further supporting a post-entry role of RAF1 during HCMV infection (see Fig. S1A through D). Similar to the treatment with pharmacological inhibitors and RAF1-shRNA, the CRISPR results suggest that RAF1 supports high-titer HCMV replication.

### Pharmacological inhibition of RAF1 attenuates HCMV TB40/E cell-to-cell spread in fibroblasts and epithelial cells

**Fig 8:**
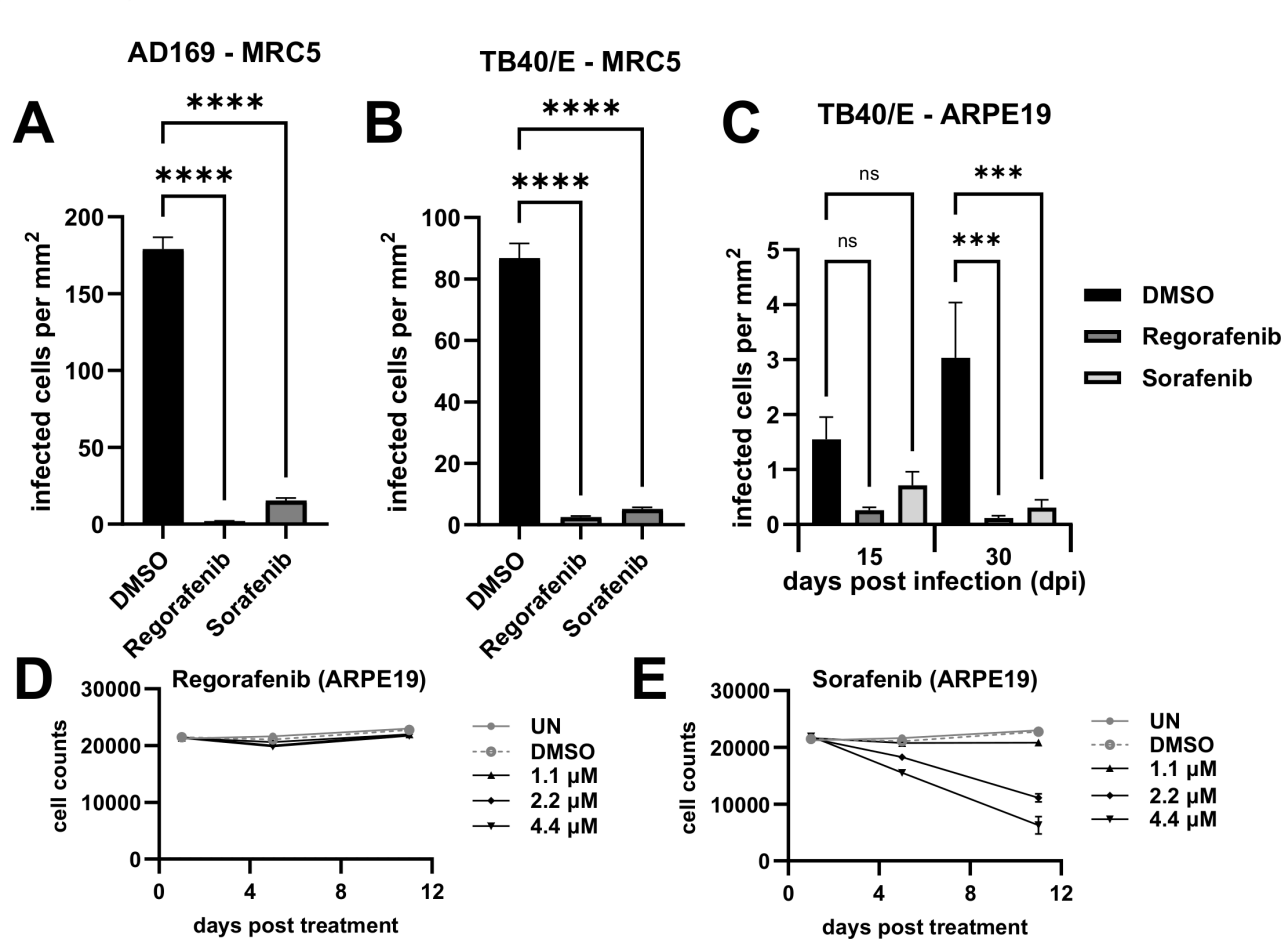
Pharmacological inhibition of RAF1 attenuates HCMV TB40/E cell-to-cell spread in fibroblasts and epithelial cells. (**A, B**) MRC5 fibroblasts or (**C**) ARPE19 epithelial cells were treated with 2.17 µM of regorafenib or sorafenib or DMSO control at the time of infection with (**A**) AD169-GFP or (**B, C**) TB40/E-mCherry at an MOI of 0.01. Viral spread was calculated by cells positive for (**A**) GFP or (**B**) mCherry on Day 12 in MRC5 cells or (**C**) mCherry on Days 15 and 30 in ARPE19 cells [avg ± standard error of the mean (SEM), *n* = 48]. Significance was established by a one-way analysis of variance test, followed by Tukey’s multiple-comparison test. (**D, E**) Mock-infected ARPE19 cells were treated with indicated concentrations of (**D**) regorafenib or (**E**) sorafenib at t = 0. Cells were fixed on Day 1, 5, or 11. Hoechst-stained nuclei were counted and plotted against time (avg ± SEM, *n* = 3).

So far, our experiments have employed the use of HCMV strain AD169. To further test the impact of RAF1 inhibition on the replication of another HCMV strain, we targeted RAF1 during TB40/E infection in fibroblasts and epithelial cells. First, viral spread was measured using the AD169-GFP virus in MRC5 fibroblasts, where inhibition of RAF1 by regorafenib and sorafenib significantly inhibited viral cell-to-cell spread (Fig. 8A). In the same cells, the cell-to-cell spread of TB40/E was also significantly reduced (Fig. 8B). Similarly, the cell-to-cell spread of TB40/E was reduced in ARPE19 epithelial cells treated with RAF1 inhibitors relative to DMSO (Fig. 8C). Cell counts in ARPE19 cells were not impacted by regorafenib treatment at multiple doses (Fig. 8D). However, increasing concentrations of sorafenib did result in lower cell numbers (Fig. 8E), suggesting that sorafenib may be more toxic to ARPE19 cells. Nonetheless, regorafenib was well tolerated, and both drugs inhibited viral infection in the ARPE19 cell line. These data suggest that RAF1 is important for multiple strains of HCMV to replicate in both fibroblasts and epithelial cells.

## DISCUSSION

A variety of viruses rely on cellular growth factor signaling for many facets of viral infection, including entry, the production of viral progeny, and the release of virions from the cell (20–24). These signaling molecules include various MAPK pathway activators and components, such as PDGFR, RAS, MEK, and ERK (23). Here, we show that RAF1 plays an important role in HCMV infection and is regulated by AMPK-mediated phosphorylation. Our data indicate that AMPK induces RAF1-specific phosphorylation during HCMV infection (Fig. 1), which in turn enhances RAF1 binding to the 14-3-3 protein, an important co-factor for RAF1 activation (Fig. 2B and C).

We find that pharmacological inhibition of RAF1 inhibits HCMV infection, reducing viral DNA accumulation and the production of viral progeny (Fig. 3 to 5). Our results are consistent with a previous report that sorafenib can inhibit HCMV infection in different cell types (36). Both sorafenib and regorafenib can inhibit multiple RAF kinases, including BRAF and RAF1, as well as VEGFR and c-Kit family members (Table 1) (50, 51). To address this issue, we targeted RAF1 more specifically via shRNA and CRISPR and found that, in both cases, HCMV infection was reduced, albeit to a smaller extent than with pharmacological treatment (Fig. 6 and 7). One possibility is that pharmaceutical-mediated RAF1 inhibition is more complete, that is, residual RAF1 activity could be present upon shRNA and CRISPR-mediated RAF1 inhibition and thus support infection to a greater extent than pharmaceutical inhibition. Another possibility for the discrepancy could be that other RAF family members may be able to support viral replication in the absence of RAF1 (e.g., ARAF or BRAF) activities that would likely be inhibited by sorafenib and regorafenib. To examine the contributions that ARAF or BRAF make toward HCMV infection, we targeted their expression via shRNA (Fig. 6). Reduced expression of either ARAF or BRAF did not inhibit infection, suggesting that RAF1 is the most important RAF kinase for productive HCMV infection in fibroblasts. However, we cannot rule out the possibility that ARAF or BRAF could be compensating for the loss of RAF1 when it is targeted via shRNA or CRISPR. Unfortunately, we have found that fibroblasts will not tolerate targeting multiple genes across multiple antibiotic selections, making elucidating potential ARAF or BRAF compensatory effects for RAF1 inactivation difficult.

While both shRNA- and CRISPR-mediated targeting of RAF1 inhibited HCMV replication, they did so to different extents, with shRNA targeting RAF1 resulting in greater inhibition of HCMV replication in comparison to CRISPR-mediated targeting. Two different shRNA sequences were employed to target RAF1, suggesting that off-target effects of a single shRNA sequence are likely not responsible for the greater inhibition. Similarly, two different guides were employed for CRISPR targeting. Other possible contributing factors could be the means of shRNA versus CRISPR delivery. The shRNA constructs were delivered by lentiviral transduction and subsequent puromycin selection. Furthermore, lentiviral transduction could potentially result in some activation of the innate immune signaling, which could impact viral replication. In contrast, CRISPR RNPs were delivered via transfection without antibiotic selection. One or more of these differences could be working as a synthetic combinatorial effect with RAF1 reduction, resulting in differences in the magnitude of viral inhibition. It is also challenging to deconvolute cellular RAF1 expression patterns from bulk western blot data. For example, puromycin selection of shRNA could yield a more homogenous reduction in RAF1 levels, as opposed to CRISPR RNP transfection in the absence of selection, which would be predicted to give rise to a mixed population of heterozygous and homozygous edited alleles that could provide greater support for viral infection. While all modes of targeting RAF1 suggested that it is important for HCMV infection, the differences in the magnitude of inhibition suggest that it can be beneficial to target gene expression in multiple ways to obtain a more accurate picture of the contributions to viral infection.

Sorafenib and regorafenib treatment inhibited HCMV replication in fibroblasts and epithelial cells (Fig. 3 and 8). Collectively, these results suggest the possibility of using these pharmacological RAF1 inhibitors to treat clinical HCMV infection. Regorafenib has FDA approval for the treatment of metastatic colorectal cancer, advanced gastrointestinal stromal tumors, and hepatocellular carcinoma. Sorafenib has FDA and European Commission approval for the treatment of hepatocellular, renal, and thyroid carcinomas. Both drugs are generally well tolerated in patients with mild side effects (52, 53). HCMV can be a significant pathological factor in cancer patients receiving immunosuppressive therapies (54–56). In this regard, the anti-viral activity of these compounds could, therefore, potentially help prevent HCMV-associated complications when they are used for cancer treatment.

We find that RAF1 phosphorylation during HCMV infection is complex. At early times post-infection, HCMV increases the levels of S338 phosphorylated RAF1, consistent with its activation (39). By 48 hours, these levels begin to fall. The total levels of RAF1 also fall over the course of infection (Fig. 1). Collectively, these data suggest that the activity of RAF1 is reduced at later times during infection. However, the functional relevance of potentially reduced RAF1 activity to infection is unclear. Of note, overexpression of RAF1 throughout infection does not negatively impact the production of viral progeny (Fig. 2), suggesting that increased expression of RAF1 at late time points does not impact HCMV infection. However, we cannot rule out the possibility that RAF1 activity is still reduced at these late time points despite RAF1 over-expression (e.g., via inhibitory phosphorylation).

RAF1 inhibition modestly lowered the accumulation of IE1 and IE2 at early times of infection and more dramatically decreased viral DNA accumulation over the course of infection (Fig. 5A through E). It is unclear if the observed reduction in IE1 or IE2 levels is responsible for the observed reduction in viral DNA replication. Future experiments will be necessary to elucidate how pharmacological targeting of RAF1 mechanistically impacts HCMV infection.

We assessed RAF1 phosphorylation using two phosphospecific antibodies that have been linked to RAF1 activity. Notably, over 50 different RAF1 phosphorylations have been described in the literature (phosphosite.org), which highlights the difficulty of making broad conclusions based on data utilizing a few phosphospecific antibodies. Potentially capturing a broader representation of RAF1 post-translational modifications, HCMV infection induced dramatic changes to the isoelectric point of the majority of migrating RAF1 species, collapsing them and shifting them to a more acidic portion of the gradient (Fig. 1F). These results are consistent with infection broadly inducing RAF1’s phosphorylation. Furthermore, treatment with Compound C substantially reversed this acidic shift, consistent with AMPK activity being important for RAF1 phosphorylation. Data using the RAF1 phospho-Ser621-specific antibody supported this possibility. HCMV infection induced RAF1 phosphorylation at Ser621, a site known to be phosphorylated by AMPK (47), and this phosphorylation was reversed with Compound C treatment (Fig. 1). These data suggest that AMPK substantially modulates RAF1 phosphorylation during HCMV infection, yet many questions remain about how these AMPK-dependent modifications might functionally contribute to infection.

We found that AMPK-dependent RAF1-Ser621 phosphorylation increases as HCMV infection progresses (Fig. 1). Studies suggest that RAF1 phosphorylation at Ser621 is required for its activation via 14-3-3 binding (40, 57). Specifically, this phosphorylation facilitates ATP-binding upon 14-3-3 interaction (44). Consistent with this, RAF1 associates with 14-3-3 during infection, which can be substantially reduced by the S621A mutation or by Compound C treatment (Fig. 2). Over-expression of RAF1-S621A did not impact HCMV infection; however, firm conclusions about the potential contributions of RAF1-Ser621 phosphorylation to infection are difficult, given that endogenous RAF1 capable of Ser621 phosphorylation was present in these experiments.

AMPK is known to be important for productive HCMV infection in part through HCMV-induced metabolic remodeling, for example, inducing GLUT4 expression and activating glycolysis (18, 19). Here, our data suggest that AMPK phosphorylates RAF1, a central MAPK signaling component that we find is important for infection. Collectively, our evidence suggests a link between HCMV-induced kinase pathways that are important for HCMV infection. Given HCMV’s reliance on these pathways, they may represent therapeutically useful vulnerabilities, but questions remain as to how the cross-talk between these pathways might contribute to infection.

## MATERIALS AND METHODS

### Cell and viral culture

Human 293T cells (ATC CCRL-3216), telomerase-expressing human foreskin fibroblasts (HFF), telomerase-expressing MRC5 fibroblast cells, and HFF or MRC5 derivative cell lines were cultured in Dulbecco’s modified Eagle medium (DMEM; Invitrogen). ARPE19 retinal epithelial cells were cultured in Dulbecco’s modified Eagle medium:nutrient mixture F12 (DMEM F12; Invitrogen). MRC5 and HFF parental cell lines were generated via lentiviral transduction of telomerase previously described here (37). DMEM and DMEM F12 were supplemented with 10% fetal bovine serum (Atlanta Biologicals or Biowest), 4.5 g/L glucose, and 1% penicillin–streptomycin (pen-strep; Life Technologies), and cells were maintained at 37°C in a 5% (vol/vol) CO_2_ atmosphere. Cell lines used for specific experiments are indicated in each figure legend.

Prior to HCMV infection, cells were grown to confluence and maintained in serum-free medium for 24 h. Cells were mock- or HCMV-infected at the indicated multiplicity of infection (MOI), with HCMV viral strains AD169, AD169-GFP, or TB40/E-mCherry as indicated in the figure legends, for an adsorption time of 120 min. The AD169-GFP virus was generated as described here (58). The TB40/E-mCherry virus was a generous gift from Christine M. O’Connor and Eain Murphy and generated as described here (59). Afterward, the viral inoculum was removed, and cells were washed one time with phosphate-buffered saline (PBS; Invitrogen) and subsequently cultured in serum-free DMEM or DMEM F12 for the duration of infection, unless otherwise indicated. For viral spread assays, cells were grown in a 384-well plate; after adsorption, the medium was not aspirated, and cells were not washed with PBS, but brought up to the final cell culture volume of 70 µL per well for the remainder of the experiment. All drug compounds, when indicated, were added at the time of viral adsorption and again with replacement medium after viral inoculum was removed for the remainder of the experiment. All viral stocks were propagated in MRC5 cells and maintained in serum-free DMEM with pen-strep present. Viral stock titers were calculated by plaque assay performed in MRC5 cells.

### Flag-RAF1 plasmid cloning

The Flag-RAF1-WT and Flag-RAF1-S621A constructs were cloned via Gibson assembly into the pLenti-puro vector. The RAF1 sequence was taken from the pDONR223-RAF1 construct ordered from Addgene, a gift from William Hahn & David Root (Addgene plasmid #23832; https://www.addgene.org/23832/; RRID:Addgene_23832). The FLAG tag was inserted with a Gibson assembly primer. The S621A mutation was introduced via Gibson assembly into the wild-type (WT) construct. Assembled constructs were heat-shocked into Stbl3 bacterial cells for plasmid propagation and storage.

### shRNA knockdown

For shRNA knockdown experiments, pLKO.1-based Mission shRNA constructs targeting the RAF proteins were obtained from the Sigma-Aldrich Mission shRNA Library (Sigma/Broad Institute). For RAF1, there is clone number TRCN_1065 (shRAF1-1) or TRCN_1068 (shRAF1-2); for ARAF, there is clone number TRCN0000000571 (shARAF-1) or TRCN0000315027 (shARAF-2); and for BRAF, there is clone number TRCN0000196844 (shBRAF-1) or TRCN0000196918 (shBRAF-2). Mission pLKO.1-puro scrambled non-targeting empty-vector (EV) control shRNA (shEV; SHC002) was used as a control in all shRNA experiments. The effectiveness of each shRNA construct to knock down the targeted protein was assessed by RT-qPCR.

### Lentiviral transductions and cell line generation

For each RAF1 lentiviral construct, three 10 cm dishes were seeded with 293T cells at 2 × 10^6^ cells per dish; similarly, one 10 cm dish was seeded for each shRNA construct. After 24 h, each dish was transfected with 2.6 µg lentiviral transfer or shRNA construct plasmid, 2.4 µg PAX2 packaging plasmid, and 0.25 µg VSV-G envelope plasmid using Fugene 6 (Promega) transfection reagent. After an additional 24 h, the medium was removed and replaced with 4 mL of fresh medium for 24 h. The following day, the supernatant was filtered through a 0.45 µm syringe filter to remove any debris and dead cells. Filtered lentiviral supernatant was introduced to MRC5 cells for FLAG-RAF1 experiments or HFF cells for shRNA experiments in the presence of 5 µg/mL polybrene (Millipore Sigma). Three hours later, the lentivirus on FLAG cells was removed, and freshly filtered lentivirus from a second dish of 293T cells was added without Polybrene. After three more hours, lentivirus was removed and replaced with freshly filtered lentivirus from the final dish of 293T cells without polybrene and incubated overnight. Plates transduced with the shRNA lentiviral constructs were left overnight in the presence of 5 µg/mL Polybrene. The following day, lentivirus was replaced with 10 mL of fresh DMEM containing 1% FBS and 1% Pen-Strep. 72 h post-transduction, cells were selected with 1 µg/mL puromycin in normal growth conditions. Selection efficiency/efficacy was monitored by cell death of mock-transduced parental cells in the presence of puromycin. Once mock cells were dead, shRNA-transduced cells were immediately used for experiments. Flag-RAF1 cells were used immediately, and some cells were frozen down for later use. If thawed, the expression of Flag-RAF1 was verified by western blot before experiments were carried out.

### CRISPR knockout cell line generation

CRISPR synthetic single guide RNAs (sgRNA) and SpCas9 2NLS nuclease were ordered from Synthego Corporation. Guide sequences are listed in Table 2. For each transfection, 30 pmol of the sgRNA and 10 pmol of Cas9 were introduced to 2 × 10^5^ MRC5 or HFF cells via the Neon Transfection System (Invitrogen) using the Neon Transfection System 10 µL kit according to the manufacturer’s protocol. Neon electroporation parameters for the MRC5 fibroblasts were as follows: voltage—1100 V, pulse width—30 ms, one pulse; and for the HFF cells: voltage—1650 V, pulse width—10 ms, three pulses. Parental cells were shocked via Neon using the same parameters, with equivalent volumes of buffer and cell suspension. After electroporation, cells were immediately plated into prewarmed DMEM with 10% FBS and no antibiotics overnight in a 12-well plate. Cells were propagated as normal once they became confluent (in the presence of 10% FBS and 1% Pen-Strep). Primers used to sequence genomic DNA for ICE analysis (Synthego Corporation) to determine the KO score of each polyclonal cell line are listed in Table 3.

**TABLE 2:** CRISPR guide sequences

Gene	Polyclonal cell line	Guide target sequence
RAF1	MRC5 KO	GCATCAATGGAGCACATACA
RAF1	HFF KO #1	GCTTGGAAGACGATCAGCAA
RAF1	HFF KO #2	GCATCAATGGAGCACATACA

**TABLE 3:** Primers used for genomic PCRs of CRISPR polyclonal knockout cell lines

Primer name		Primers 5′ to 3′
RAF1_gPCR_5_for	Forward	TTTAACTGCTGGTCCCATTTTCC
RAF1_gPCR_5_rev	Reverse	AGTAGTTGGAAAGGCCTAGAGAC
RAF1_gPCR_6_for	Forward	TCTTTTAACTGCTGGTCCCAT
RAF1_gPCR_6_rev	Reverse	AGTAGTTGGAAAGGCCTAGAGA

### Drugs and compounds

All drugs were suspended in vehicle control, dimethyl sulfoxide (DMSO; Corning). Compound C (CC) (Millipore Sigma or MedChemExpress) was purchased in solution at a concentration of 10 mM in DMSO and used at a concentration of 5 µM for all experiments. Regorafenib (Reg) and sorafenib (Sor) (MedChemExpress) were suspended in DMSO and used at a concentration of 2.17 µM, unless otherwise indicated. Hoechst 33342 (Invitrogen) was purchased in solution at a concentration of 10 mg/mL and diluted 1:2,000 in PBS for use. Puromycin (Millipore Sigma) was purchased as a powder, suspended to 10 mg/mL in water, and used at a final concentration of 1 µg/mL for all lentiviral transduction selections.

### Immunoblotting

Cells were harvested by washing once with cold PBS, then scraping on ice in 1× RIPA (10× RIPA: 0.5 M Tris–HCl, pH 7.4, 1.5 M NaCl, 10 mM EDTA, 2.5% deoxycholic acid, 10% Triton X-100) or cold lysis buffer [20 mM Tris–HCl (pH 7.5), 100 mM NaCl, 1 mM MgCl2, 1% IGEPAL CA-630, 1 mM EDTA] containing a protease inhibitor tablet (Pierce; A32955) and PhosSTOP tablet (Sigma) per 10 mL of buffer. RIPA was used to harvest all western blot samples, with the exception of the RAF1 overexpression experiments, where lysis buffer was used instead for the pulldowns. Scraped cells in lysis buffer were incubated in a tube on ice for 20 min and sonicated, and then the insoluble fraction was pelleted and discarded. Soluble proteins were quantified via Bradford assay (BioRad), and samples were normalized to the total protein concentration and added three-part protein to one-part 4× disruption buffer (200 mM Tris–HCl (pH 7.0), 11% sucrose, 20% beta-mercaptoethanol, 8% SDS), and boiled for 5 min. Samples were spun down prior to running on a gel.

For FLAG pulldown experiments, protein lysates were added to 50 µL of prewashed Anti-FLAG M2 affinity gel beads (Sigma) and incubated at 4°C overnight with gentle tumbling. Pulldowns were washed four times with modified lysis buffer (IGEPAL CA-630 reduced to 0.1%). Then 1× disruption buffer was added to the washed beads and boiled for 5 min. Samples were spun down, and the disruption buffer containing immunoprecipitated proteins was transferred to a new tube.

Proteins were separated on a 10% SDS–PAGE gel in Tris–glycine running buffer. Samples were transferred to a nitrocellulose membrane in Tris–glycine transfer buffer and stained with Ponceau S to visualize proteins and ensure equal loading. Blots were blocked with 5% dried non-fat milk in Tris-buffered saline-0.1% Tween 20 (TBST), followed by primary and secondary incubations in 5% bovine serum albumin in TBST (Flag primary was diluted in 2.5% milk in TBST). Antibodies were used at concentrations recommended by the manufacturer and are listed in Table 4. Blots were developed with enhanced chemiluminescence (ECL, Bio-Rad) and imaged using a Molecular Imager Gel Doc XR+ System (Bio-Rad). Western blot densitometry was measured using Bio-Rad Image Lab Software using the total band intensity for quantification analysis. Graphs showing western blot quantification represent the average density ± standard error of two independent experiments.

**TABLE 4:** List of antibodies used^*a*^

Antibody	Source	Catalog number
GAPDH (D16H11)	Cell Signaling Technology	5174
Phospho-Raf1 (c-Raf) (Ser338)	Cell Signaling Technology	9427
Phospho-Raf1 (S621)	Abcam	ab4767
Phospho-Raf1 (c-Raf) (Ser621)	Thermo Fisher Scientific	44–504G
Raf1 (c-Raf) (D4B3J)	Cell Signaling Technology	53745
Phospho-MEK1/2 (Ser217/S221)	Cell Signaling Technology	8211
MEK1/2 (47E6)	Cell Signaling Technology	9126
Phospho-p44/42 MAPK, P-ERK1/2 (Thr202/Tyr204)	Cell Signaling Technology	9101
p44/42 MAPK, ERK1/2	Cell Signaling Technology	9102
DYKDDDDK Tag (D6W5B) Rabbit	Cell Signaling Technology	14793
pan-14-3-3 (E9S9M)	Cell Signaling Technology	95422
AMPKa2	Cell Signaling Technology	2757
IE1	(60)	*
IE2	(60)	*
UL44	Virusys Corporation	CA006
pp28	(61)	*
UL26	(62)	
Goat anti-rabbit secondary	Bio-Rad	1706515
Goat anti-mouse secondary	Bio-Rad	1706516

^*a*^ *, generous gift from Thomas Shenk.

### 2D gel electrophoresis

Cells were treated with Compound C and infected in 15 cm dishes, then 2D gel samples were collected by scraping cells in 250 µL of 2D rehydration buffer [4% CHAPS, 8 M urea, 0.5% IPG buffer (pH 3.0–10.0; GE Healthcare), 0.002% bromophenol blue, and 40 mM DTT (DTT was added fresh before extraction)]. Samples were spun to pellet insoluble fraction and loaded on a 13 cm Immobline Drystrip isoelectric-focusing gel (pH 3.0–10.0; GE Healthcare), and proteins were separated in the first dimension using the IPGphor isoelectric-focusing system (Amersham Pharmacia Biotech) following the manufacturer’s protocol. Then, the gel was equilibrated for 15 min in SDS equilibration buffer [6 M urea, 75 mM Tris–HCl (pH 8.8), 30% glycerol, 2% SDS, 0.002% bromophenol blue], and proteins were run in the second dimension by SDS-based electrophoresis on a 10% polyacrylamide gel. Finally, samples were treated as a typical western blot by transferring the proteins to a nitrocellulose membrane, blocking with milk, and immunoblotting for RAF1, as described above.

### Cell-to-cell spread assay and nuclear counts

For the drug titrations performed in Fig. 3, cells were seeded using the Multidrop Combi Reagent Dispenser (Combi; Thermo Scientific) into a 384-well plate and grown to confluence. The medium was then aspirated and replaced with serum-free medium for 24 h. Then, cells were treated with regorafenib and sorafenib using the D300 digital dispenser (D300; Hewlett-Packard Company, L.P.) with accompanying software to titrate into the low nM range. Immediately afterwards, AD169-GFP virus was dispensed using the Combi. After 2 h, all wells were brought up to the final culture volume of 70 µL with fresh medium and the drug for the remainder of the experiment.

For cell spread assays in Fig. 7D, E , 8A through C, indicated cell lines were seeded into 384-well plates and grown to confluence. The medium was then aspirated and replaced with serum-free medium for 24 h. Then, cells were treated with 2.17 µM drug or volume equivalent of DMSO control (using the VOYAGER automated digital pipette; Integra Biosciences) when indicated (Fig. 8) and immediately infected with AD169-GFP or TB40/E-mCherry, as indicated, using the Voyager pipette for 2 h. All wells were brought up to the final volume of 70 µL with drug or DMSO control for the remainder of the experiment.

GFP- or mCherry-positive cells were imaged on the Cytation 5 imaging reader and masked using the accompanying Gen5 software (Agilent Technologies, formerly BioTek Instruments, Inc.). One image from each well was taken and quantified, representing an individual infection and replicate. The final cell-to-cell spread was calculated as GFP- or mCherry-positive cells per area of the entire well. Cell counts in Fig. 4 and Fig. 8D and E were acquired by Hoechst 33342 (Invitrogen) staining the nuclei of treated cells, followed by imaging on the Cytation 5 imaging reader. Nuclei were counted using Gen5 software. Statistics presented in these experiments represent the average cell-to-cell spread or cell count ± standard error at each point on the graph.

### Assessing viral titers

When indicated, TCID50 was used to assess experimental viral titers. Alternatively, a high-throughput method was developed in our laboratory where infectious units per mililiter (IU/mL) were measured using a GFP-expressing virus. Experimental viruses were serially diluted using the Assist Plus pipetting robot (Integra Biosciences) across a 384-well plate of confluent MRC5 cells. Plates containing serially diluted viruses were incubated for 48 h. Prior to imaging, cellular medium was exchanged for FluoroBrite DMEM (Gibco) medium for higher-quality imaging. GFP was measured using the Cytation 5 imaging reader. Viral titers were then calculated based on 10-fold dilutions allowing for a higher number of replicates to be titered at one time. Viral titer figures are presented as either bar or line graphs representing the average viral titer ± standard error.

### Real-time quantitative PCR

RNA was extracted using Trizol reagent (Invitrogen) according to the manufacturer’s protocol. RNA was treated with DNase I (Invitrogen) and used to synthesize cDNA using random hexamer primers (Invitrogen) and Superscript II reverse transcriptase (Invitrogen). Quantitative PCR (qPCR) was carried out using Fast SYBR Green Master Mix (Applied Biosystems), a 7500 fast real-time PCR system (Applied Biosystems), and StepOne real-time PCR software (Applied Biosystems). Primers used for each reaction are listed in Table 5. Primers were obtained from PrimerBank, ID:189458830c2 for RAF1, ID:371875962c1 for ARAF, and ID: 187608632c2 for BRAF (63). Relative RNA levels for RAF1, ARAF, and BRAF were measured and normalized to GAPDH levels, and then indicated shEV samples using the 2-ΔΔCT method. Statistical analysis of shRNA knockdown represents the average RNA levels ± standard error.

**TABLE 5:** List of qPCR primer sequences

Gene		Sequence (5′ to 3′)
RAF1	Forward	CCGAACAAGCAAAGAACAGTG
	Reverse	GACGCAGCATCAGTATTCCAAT
ARAF	Forward	GGATGGCATGAGTGTCTACGA
	Reverse	GGTCAGCGGGACATCTTCA
BRAF	Forward	TGGGGAACGGAACTGATTTTTC
	Reverse	TTTTGTGGTGACTTGGGGTTG
GAPDH	Forward	CATGTTCGTCATGGGTGTGAACCA
	Reverse	ATGGCATGGACTGTGGTCATGAGT
IE1	Forward	GGAGGAGCATCCGTGACCTC
	Reverse	GAAACATGCGGCTCACCTCG

### Viral DNA quantification

Viral DNA (vDNA) was extracted by scraping cells from a 12-well dish into 150 µL of QuickExtract DNA Extraction Solution (LGC Biosearch Technologies) following the manufacturer’s protocol and using a thermal cycler to control temperature steps. DNA was serially diluted at 1:1000 in deionized water, and equal amounts of sample were assessed via RT-qPCR (primers are listed in Table 5). Relative levels of IE1 were measured and normalized to the highest CT value (sorafenib at 48 h post infection) using the 2-ΔCT method. Statistics are presented as the average area under the curve (AUC) ± standard error, compared by a one way analysis of variance (ANOVA), followed by Tukey’s multiple-comparison test.

### Statistical analysis

Statistical analysis was carried out using GraphPad Prism 9 statistical software, sample number (n) ± standard deviation (STD), or standard error mean (SEM) are plotted as indicated in figure legends. A one-way ANOVA, followed by Tukey’s multiple-comparison test or Student’s *t*-test was performed, as indicated in the figure legends. *P* < 0.05 was considered statistically significant, where * is *P* < 0.05, ** is *P* < 0.01, *** is *P* < 0.001, and “ns” indicates not significant. The area under the curve (AUC) was calculated using GraphPad Prism 9 software. IC50 values reported in Fig. 3 were calculated using GraphPad Prism 9 using the nonlinear regression (curve fit), [Inhibitior] vs. response—variable slope (four parameters).

## Data Availability

Data to support our findings are included in the article.
